# Impact of COVID-19 on food insecurity using multiple waves of high frequency household surveys

**DOI:** 10.1038/s41598-022-05664-3

**Published:** 2022-02-03

**Authors:** Shouro Dasgupta, Elizabeth J. Z. Robinson

**Affiliations:** 1 Centro Euro-Mediterraneo sui Cambiamenti Climatici (CMCC), Venice, Italy; 2 RFF-CMCC European Institute on Economics and the Environment (EIEE), Centro Euro-Mediterraneo sui Cambiamenti Climatici, Venice, Italy; 3 Università Ca’ Foscari Venezia, Venice, Italy; 4 Grantham Research Institute on Climate Change and the Environment, London School of Economics and Political Science (LSE), London, UK

**Keywords:** Risk factors, Sustainability

## Abstract

In response to the rapid spread of COVID-19, governments across the globe have implemented local lockdowns that have led to increased unemployment and have disrupted local and international transport routes and supply chains. Whilst such efforts aim to slow or stop the spread of the SARS-CoV-2 virus, they have also resulted in increased food insecurity, whether due to reduced incomes or increased food prices. This is the first paper to track food insecurity and its determinants during the pandemic using multi-country and multi-wave evidence. Using data from 11 countries and up to 6 waves of High-Frequency Phone Survey data (household-level surveys) on COVID-19 and its impacts, we use a fixed-effects linear probability model to investigate the socioeconomic determinants of food insecurity during the pandemic for each country using household-level data over multiple waves. We control for socioeconomic characteristics including gender and education of the household head; income and poverty status of the households during the pandemic; safety nets in the form of cash and food assistance; coping strategies adopted by households; and price effects of major food items. Our findings suggest that cash safety nets appear to have been more effective than food in terms of reducing food insecurity during the pandemic; and that those particularly hard hit are female headed-households (highest in Malawi: 0.541, 95% CI 0.516, 0.569; lowest in Cambodia: 0.023, 95% CI 0.022, 0.024), the less educated (highest in Djibouti: − 0.232, 95% CI − 0.221, − 0.244; lowest in Nigeria: 0.006, 95% CI − 0.005, − 0.007), and poorer households (highest in Mali: 0.382, 95% CI 0.364, 0.402; lowest in Chad: 0.135, 95% CI 0.129, 0.142). In line with the existing literature, our results show that, even controlling for income loss and poverty status, those households who had to borrow rather than rely on savings had a higher probability of suffering from food insecurity. Distinct differences in the efficacy of safety nets across the 11 countries, and the differential impact of the pandemic on different groups within societies, suggest in-depth country-specific studies are needed to understand why some countries have coped better than others. Our paper highlights the importance of improving household resilience to future systemic crises, and using evidence-based best practice in the design of relevant policy instruments.

## Introduction

The COVID-19 pandemic triggered a systemic crisis across the globe. In addition to the direct impact of the virus on people’s health, local lockdowns, recommendations to self-isolate, and disrupted travel and trade routes, have resulted in people losing their jobs, food shortages, and food prices increasing^1,2^. This perfect storm of reduced access to, and availability and affordability of, food has resulted in increased food insecurity (defined as existing when “all people, at all times, have physical and economic access to sufficient, safe and nutritious food that meets their dietary needs and food preferences for an active and healthy life”^3^, in both higher and lower-income countries^4–9^.

**Figure 1:**
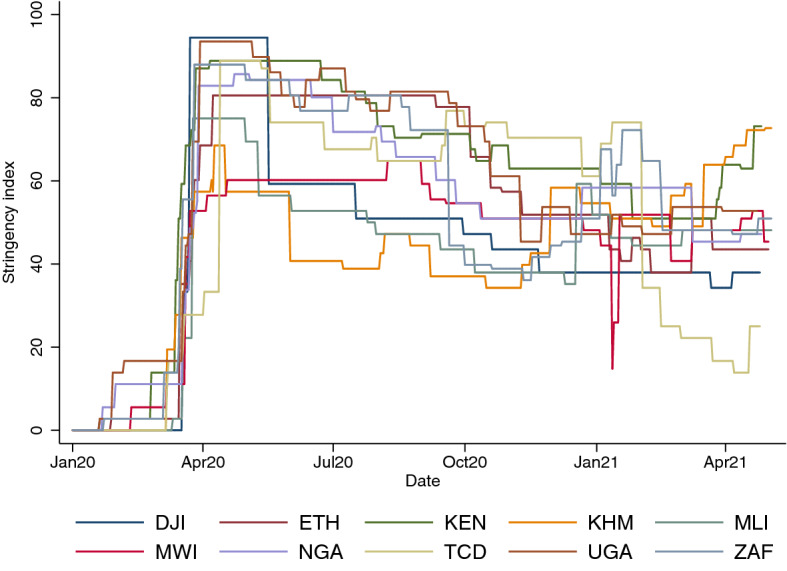
Stringency of government responses according to OxCGRT.

In this paper, taking advantage of multiple waves of High Frequency Phone Surveys (HFPS) of households undertaken since the pandemic started, linked to ongoing panel micro studies, we undertake the first multi-country, multi-period analysis of the evolution of food insecurity in lower-income countries during the COVID-19 pandemic. Each of the governments of the 11 countries included in our analysis has implemented restrictions that include school closures, travel restrictions, and bans on public gatherings, to contain the spread of the virus^10^. While the stringency of the government responses were highest during March and April 2020 (in the early stages of the pandemic), some countries such as Djibouti and Mali started to relax their restrictions around July 2020. However, most of the sample countries continued to impose at least some restrictions until the end of 2020. With the rise of COVID-19 cases in the beginning of 2021 (Figure 1 in the Appendix), a number of countries re-imposed restrictions (Fig. 1). Countries have also implemented policies aimed at reducing the negative impacts of the pandemic and the restrictive policies, including emergency investments in healthcare facilities, and new forms of social welfare provision. We particularly focus on the policy interventions undertaken by governments to try to reduce the impact of the pandemic on food insecurity, including the use of food and cash safety nets.

Social safety nets have long been implemented by governments to protect poor and vulnerable people and improve socioeconomic conditions, and have been highlighted as being particularly important for vulnerable populations affected by negative shocks^7,11,12^. Government-funded safety nets are common across sub-Saharan Africa, indeed they can be found in every African country^13^. The explicit link between safety nets and food security, whether in higher or lower-income countries, is well documented in the literature^14^. Though there is a multitude of different safety nets, two common safety nets employed across many countries are cash transfer programmes and food-based programmes that can variously include providing food, food stamps, food vouchers, and food for work programmes^13^.

A key debate in the literature, and among policy circles, is the extent to which cash or food assistance has most impact in reducing food insecurity^15^. Naturally, public nutrition programmes and food safety nets more broadly are designed to directly reduce food insecurity. Yet from a utility-maximising perspective, cash transfers tend to be considered to be more economically efficient because, at the margin, they do not distort consumption and production choices^16–18^. Further, cash transfers are often cheaper administratively^18^. There are also non-economic reasons for preferring cash to food transfers, such as that they are less “paternalistic”^15,19^.

Despite theoretical and non-economic rationales for preferring cash to food transfers, there is a considerable body of empirical evidence that, in higher-income countries, in-kind transfers such as food stamps improve household food consumption more than cash transfers^15^. In lower-income countries, increasingly researchers are using randomised controlled trials, experiments, and regression analysis, to explore empirically whether cash or in-kind transfers are most effective at reducing food insecurity. As such, contextual evidence takes precedence over theoretical underpinnings.

Our empirical findings suggest that safety nets in the form of cash assistance are more likely to reduce the chances of food insecurity among households over time. However, food assistance fails to significantly reduce the probability of food insecurity. Further, households with female and/or relatively less-educated heads, and those that are poorer or experiencing a pandemic-induced loss of income, have a higher chance of suffering food insecurity. We also show that households that were unable to rely on their savings and had to borrow to make ends meet were also more likely to suffer from food insecurity during the pandemic. As such, our paper shows the importance of understanding the socioeconomic underpinnings of COVID-19 impacts. Furthermore, heterogeneities in findings across countries reveal the need for evidence-based policies tailored to local contexts.

## Methods

### Data

In collaboration with international agencies including the European Commission, World Bank, and USAID, High Frequency Phone Surveys (HFPS) to investigate the socioeconomic impacts of the pandemic have been conducted in a number of countries (https://microdata.worldbank.org/index.php/catalog/hfps). Many of these surveys are linked to ongoing household surveys and aim to collect information on loss of employment and income, health and wellbeing, impact on education of children, food (in)security, coping strategies adopted, and safety net disbursements. Summaries of these datasets, that we use in this study, are provided in Table 1.

**Table 1:** Countries and waves.

Country	Waves conducted	Waves used	Number of households
Armenia	1	1	1648
Cambodia	4	3	1364
Chad	3	3	1748
Djibouti	2	2	1486
Ethiopia	6	6	3300
Kenya	3	3	4457
Malawi	7	6	1729
Mali	5	4	1809
Nigeria	9	4	1950
South Africa	3	3	7074
Uganda	4	4	2259

We use these high-Frequency Phone Survey data on COVID-19 to track the impacts of the pandemic within and across countries. We present and analyse data from eleven countries. Specifically, we look at the following four indicators of household food insecurity from the survey questionnaire:

1. Have you or any other adult in your household had to skip a meal in the last 30 days?
2. Did the household go without eating for a whole day in the last 30 days?
3. Did you or any other adult in your household run out of food in the last 30 days?
4. Were you or any other member in your household hungry but did not eat in the last 30 days?

Although there is considerable consistency across countries with respect to the indicators of food insecurity, coverage does vary from country to country. Whereas, for example, Malawi and Nigeria, at the time of writing, had each conducted seven waves, Armenia had only conducted one. Further, within these waves, some countries have consistently asked all four dimensions of food insecurity, whereas others have either only asked a subset, or have not asked the same questions consistently across waves.

### Estimation strategy

We use a fixed-effects linear probability model (Equation 1) to investigate the socioeconomic determinants of food insecurity during the pandemic for each country using household-level data over multiple waves for each of the food insecurity questions:

1 $$\begin{aligned} y_{it} = \delta X_{it} + \phi Z_{it} + \theta C_{it} + \alpha _{i} + \gamma _{t} + \epsilon _{it} \end{aligned}$$

where $$ y_{it}$$ is a binary outcome for household *i* in wave *t* in response to a specific food insecurity related question. $$\delta X_{it}$$ is a vector of socioeconomic and demographic characteristics such as education and gender, loss of household income compared to pre-pandemic levels/poverty status of households in the case of Chad, Djibouti, and Mali (income data are not available for these countries). In terms of safety nets, we control for whether a household has received cash or food benefits and, where this breakdown is unavailable, we control for whether a household has benefited from any safety net ($$\phi Z_{it}$$ is a vector of safety nets). To investigate whether the impact of safety nets on the probability of households suffering from food insecurity varies over time, interaction terms between the safety net variables and wave dummies are included. Because affordability of food is likely to be a strong determinant of food security, we include an additional dummy variable indicating whether the prices of major food items increased during the pandemic. We include two interaction terms; (1) increase in price and loss in income, and (2) increase in price and poverty status, to investigate whether increases in prices of major food items have a differential impact on food insecurity. $$\theta C_{it}$$ is a vector controlling for coping strategies that households have adopted during the pandemic: reliance on savings; and borrowing from friends, family, non-government organisations. Finally, we include sub-national and wave fixed-effects to control for unobserved heterogeneities (such as seasonality) across space and time. We use the sampling weights reported in the surveys.

## Results

### Government restrictions and consequent disruptions

The Oxford COVID-19 Government Response Tracker (OxCGRT) reports stringency of governments’ policies and interventions to contain the spread of the SARS-CoV-2 virus. Such measures might variously include school closures, travel restrictions, bans on public gatherings, emergency investments in healthcare facilities, new forms of social welfare provision, contact tracing and other interventions, and augmentation of health systems^10^. The index of stringency of these measures during the time period that the surveys in this paper were conducted is presented in Fig. 1. A clear pattern emerges. All 11 countries included in this paper rapidly transitioned from no restrictions at the start of 2020 to very high levels of restrictions by April 2020. Though stringency measures started to be relaxed from May 2020, most of the countries continued to impose considerable restrictions throughout the time period in consideration. Government stringency measures likely affected food security through a number of different channels, including loss of income where household members could not go to work^20^; and increasing food prices due to disruption of supply chains^21^, both of which we control for in our econometric analysis. The OxCGRT index is only available at the country-level, and not at the sub-national level. However, we control for sub-national heterogeneity in government restrictions by including regional fixed-effects and the changes in the stringency measures through the wave fixed-effects.

### Food insecurity and safety nets during the pandemic

**Figure 2:**
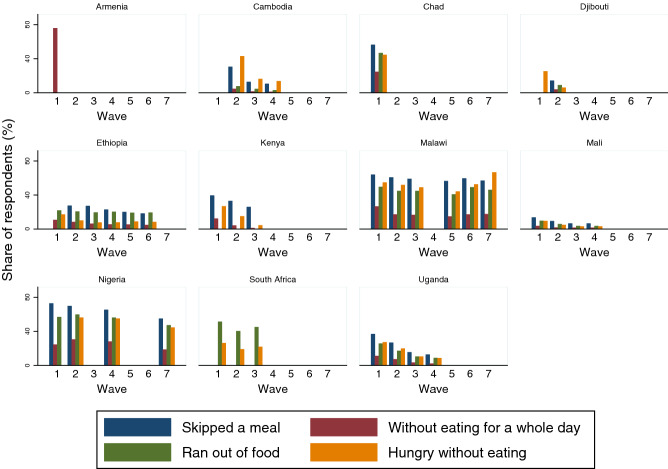
Tracking food insecurity during the pandemic.

**Figure 3:**
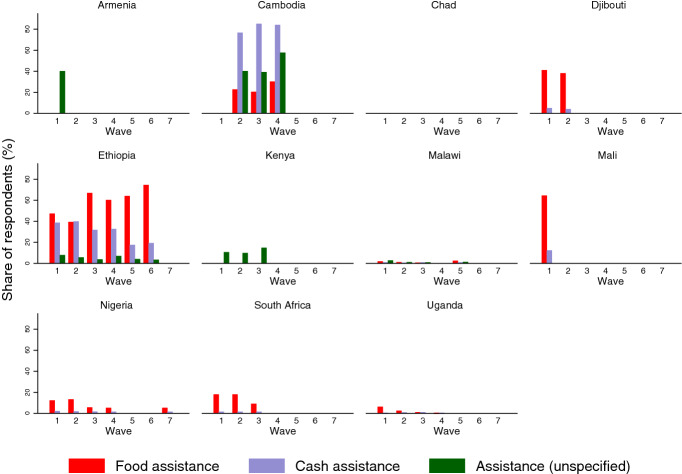
Tracking food insecurity and safety nets during the pandemic.

**Figure 4:**
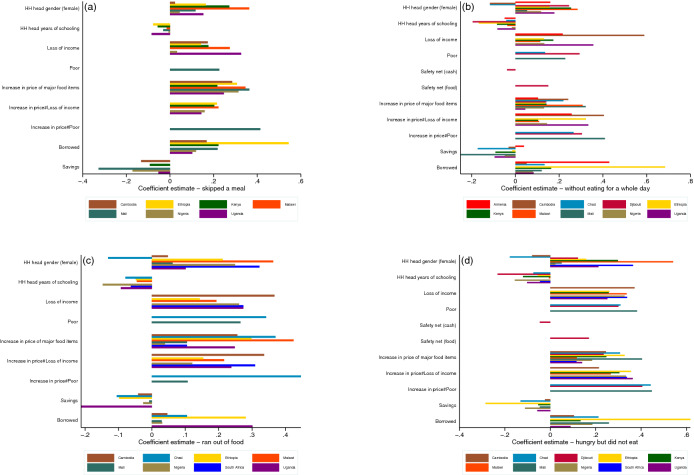
Regression coefficients—showing only statistically significant estimates; (**a**) impact on the probability of skipping a meal, (**b**) impact on the probability of eating for a whole day, (**c**) impact on the probability of running out of food, (**d**) impact on the probability of being hungry.

**Figure 5:**
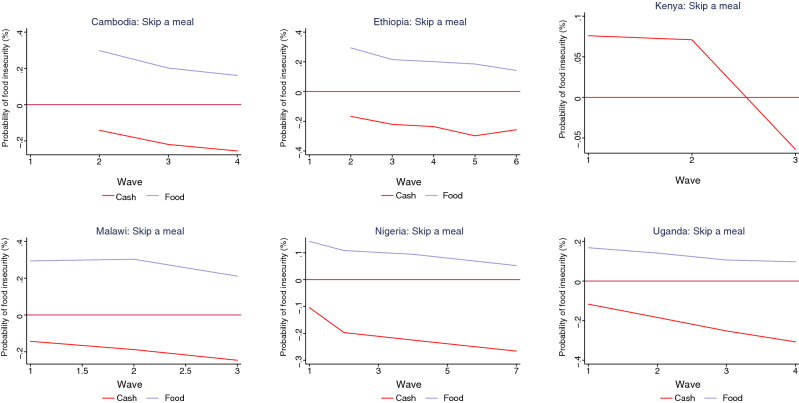
Impact of safety nets on food insecurity (skipped a meal) by country.

**Figure 6:**
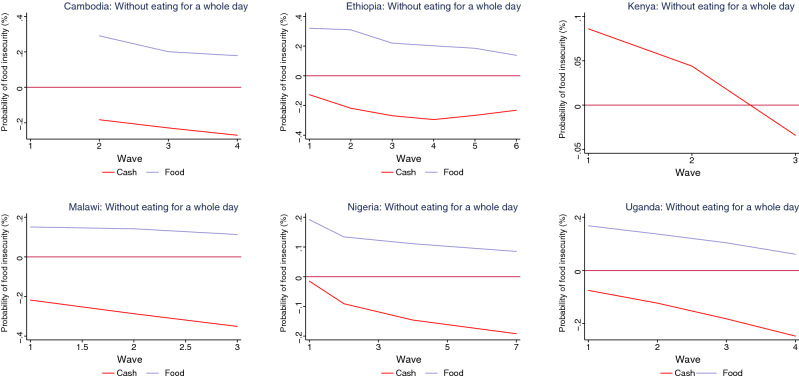
Impact of safety nets on food insecurity (without eating for a whole day) by country.

**Figure 7:**
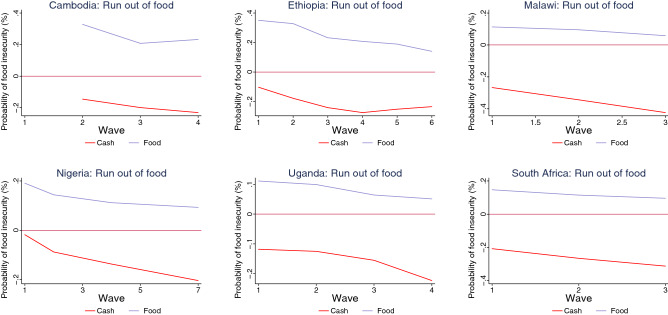
Impact of safety nets on food insecurity (run out of food) by country.

**Figure 8:**
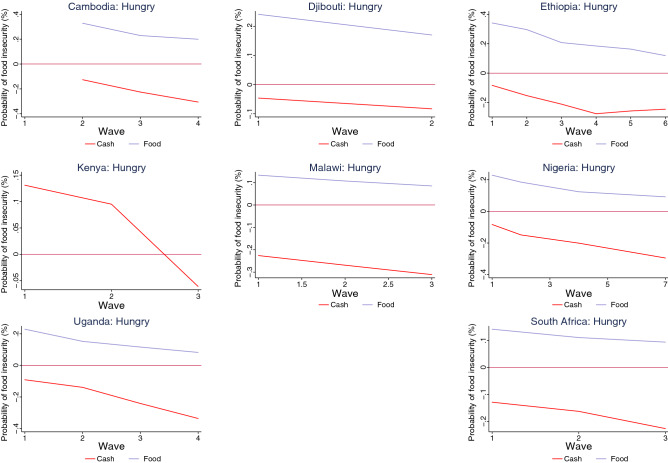
Impact of safety nets on food insecurity (went hungry) by country.

The data from the multiple waves demonstrate how food insecurity has evolved during the pandemic. We present key descriptive statistics for each of the countries (Fig. 2). Some countries with multiple waves of data reveal clear trends across time. For example, in Nigeria and Uganda, across multiple dimensions, food security appears to be improving over time. In contrast, in Malawi, South Africa, and Chad, there is no clear pattern of improvement. These national level data hide sub-national trends, the richness of which are revealed in the country maps (Figures 3–10 in the Appendix).

Whilst most countries report whether assistance is cash or food, two, Kenya and Armenia, only report government assistance in general terms, and some countries report three different categories of assistance. These discrepancies not withstanding, the data show that the extent and nature of the government safety nets varies considerably across countries (Fig. 3). Some countries, such as Ethiopia, have provided the majority of their population with either cash or food assistance. In other countries, such as Malawi, few households have received any type of government assistance. It should be noted that Chad and Mali have relatively low coverage of safety nets, this may be a government failure or it may be the case that coverage of safety nets is not being tracked. In the next section we undertake a series of econometric analyses to determine the extent to which these safety nets, in addition to other key variables, may have influenced how food insecurity has evolved during the pandemic.

### Determinants of food insecurity

The econometric results are consistent across the four indicators of food insecurity (panels (a)–(d) in Fig. 4 and Tables 2, 3, 4, 5). Our results show that female-led households generally have a higher chance of food insecurity compared to male-led households. This effect is particularly pronounced in Malawi (for example, skipping a meal: 0.284, 95% CI 0.270, 0.299; going hungry: 0.541, 95% CI 0.516, 0.569). However, there are two exceptions: In Cambodia, having a female head reduces the probabilities of households going without eating for a whole day (panel (b); Fig. 4) and going hungry (panel (d); Fig. 4); and Chad, running out of food (panel (c); Fig. 4) and going hungry (panel (d); Fig. 4). The findings also suggest that households with relatively higher educated heads have a lower probability of suffering from food insecurity, suggesting that education continues to play an important role even during a pandemic. The influence of education varies considerably, with the highest benefits with respect to reducing the chances of being “hungry but did not eat” in Djibouti (− 0.232, 95% CI − 0.221, − 0.244), Ethiopia (− 0.156, 95% CI − 0.148, − 0.164), and Nigeria (− 0.156, 95% CI − 0.149, − 0.164); and the lowest in Malawi (− 0.006, 95% CI − 0.005, − 0.007).

Food security is heavily influenced by a household’s ability to afford food. During the COVID-19 pandemic, a significant portion of the households experienced a loss in income, while prices of major food items have increased due to supply side constraints^1,2^. Our findings show that both these changes have increased households’ probability of suffering from food insecurity. The income effect is particularly strong in Cambodia (highest increase for the probability of “going without eating for a whole day”: 0.589, 95% CI 0.561, 0.619), Malawi (highest increase for the probability of “skipping a meal”: 0.274, 95% CI 0.260, 0.289), and Uganda (highest increase for the probability of “going without eating for a whole day”: 0.356, 95% CI 0.339, 0.374). For Chad, Djibouti, and Mali, we have poverty status of households rather than income information. For this group of countries, poverty status has the highest impact on the chances of being “hungry but did not eat” in Mali (0.382, 95% CI 0.364, 0.402), and the lowest in Malawi (0.135, 95% CI 0.129, 0.142). As would be expected, relatively poorer households have higher chances of suffering from food insecurity.

According to our results, increases in prices of major food items have significantly affected food security among households across all 11 countries. Households in Ethiopia were particularly affected with respect to increased chance of “going hungry but did not eat” (0.327, 95% CI 0.311, 0.344); and those in Malawi with respect to increased chance of “running out food” (0.424, 95% CI 0.404, 0.444). Both interaction terms, loss of income and price increase dummy, and poverty status and price increase dummy, suggest that households who suffered income loss and relatively poorer households experienced relatively higher increases in the probability of food insecurity due to increases in prices of major food items (the highest effect is found in Mali in terms of increasing the chances of “going hungry but did not eat”: 0.447, 95% CI 0.426, 0.470). Though expected, these findings provide empirical evidence that increases in the prices of food items have a disproportionately higher impact on poorer households.

In terms of coping strategies, our findings show that households that were able to draw down on savings during the pandemic had a lower probability of suffering from food insecurity. Savings were more effective in reducing the probability of “skipping a meal” in Mali (− 0.327, 95% CI − 0.310, − 0.343), even controlling for poverty status of the households, than in other countries. In contrast, households that had to borrow (whether from friends or family or other sources) had a higher chance of experiencing food insecurity. These findings have implications for inequality and distributional impacts of the pandemic, as relatively higher income households are likely to have sufficient savings while poorer households are are likely have to borrow to make ends meet.

#### Differential impacts of safety nets

Our findings indicate that cash transfers have been more effective than food transfers in reducing the probability of food insecurity during the pandemic (Figs. 5, 6, 7, 8). The interaction terms between the safety net dummy variables and wave dummies further suggest that the efficacy of cash transfers improved during the pandemic. For households who received food as a safety net, however, though their probability of suffering from food insecurity also declined, across all countries with relevant data, it remained positive. For Kenya, where we do not have separate information on whether assistance is in the form of cash or food transfers, the results suggest that while safety nets were ineffective in the first wave, they did reduce the probability of food insecurity among household in the second and the third waves (Figs. 5, 6, 7, 8 and Tables 2, 3, 4, 5).

**Table 2:** Determinants of food insecurity—skipped a meal.

	(1)	(2)	(3)	(4)	(5)	(6)	(7)
	Cambodia	Ethiopia	Kenya	Mali	Malawi	Nigeria	Uganda
HH head gender (female)	0.023	0.164	0.272	0.117	0.363	0.044	0.153
	(0.022, 0.024)	(0.156, 0.173)	(0.258, 0.286)	(0.111, 0.123)	(0.345, 0.381)	(0.042, 0.046)	(0.145, 0.161)
HH head years of schooling		− 0.077	− 0.056	− 0.031	− 0.015	− 0.006	− 0.084
		(− 0.073, − 0.081)	(− 0.053, − 0.059)	(− 0.029, − 0.032)	(− 0.014, − 0.016)	(− 0.005, − 0.007)	(− 0.08, − 0.088)
Income loss	0.173	0.142	0.176		0.274	0.032	0.326
	(0.164, 0.182)	(0.135, 0.148)	(0.167, 0.186)		(0.260, 0.289)	(0.031, 0.033)	(0.310, 0.342)
Poor				0.226			
				(0.215, 0.238)			
Safety net (cash)—wave 1					− 0.143	− 0.104	− 0.117
					(− 0.137, − 0.149)	(− 0.100, − 0.108)	(− 0.112, − 0.122)
Safety net (cash)—wave 2	− 0.142	− 0.167			− 0.188	− 0.197	− 0.184
	(− 0.137, − 0.146)	(− 0.159, − 0.175)			(− 0.180, − 0.196)	(− 0.188, − 0.206)	(− 0.175, − 0.193)
Safety net (cash)—wave 3	− 0.220	− 0.221			− 0.246		− 0.252
	(− 0.215, − 0.225)	(− 0.211, − 0.231)			(− 0.235, − 0.257)	− 0.225	(− 0.241, − 0.263)
Safety net (cash)—wave 4	− 0.256	− 0.236				(− 0.214, − 0.236)	− 0.307
	(− 0.248, − 0.263)	(− 0.227, − 0.245)					(− 0.294, − 0.320)
Safety net (cash)—wave 5		− 0.298					
		(− 0.285, − 0.311)					
Safety net (cash)—wave 6		− 0.257					
		(− 0.244, − 0.270)					
Safety net (cash)—wave 7						− 0.266	
						(− 0.254, − 0.278)	
Safety net (food)—wave 1					0.294	0.142	0.168
					(0.281, 0.307)	(0.137, 0.147)	(0.160, 0.176)
Safety net (food)—wave 2	0.298	0.294			0.303	0.108	0.141
	(0.284, 0.312)	(0.280, 0.308)			(0.291, 0.315)	(0.104, 0.112)	(0.134, 0.148)
Safety net (food)—wave 3	0.202	0.215			0.211		0.106
	(0.193, 0.211)	(0.205, 0.225)			(0.202, 0.220)	0.094	(0.101, 0.111)
Safety net (food)—wave 4	0.161	0.201				(0.091, 0.097)	0.097
	(0.154, 0.167)	(0.193, 0.209)					(0.093, 0.103)
Safety net (food)—wave 5		0.185					
		(0.177, 0.193)					
Safety net (food)—wave 6		0.142					
		(0.135, 0.149)					
Safety net (food)—wave 7						0.052	
						(0.05, 0.054)	
Safety net (overall)—wave 1			0.076				
			(0.074, 0.078)				
Safety net (overall)—wave 2			0.071				
			(0.069, 0.073)				
Safety net (overall)—wave 3			− 0.064				
			(− 0.062, − 0.066)				
Increase in price of major food items	0.285	0.307	0.217	0.364	0.346	0.314	0.247
	(0.271, 0.300)	(0.292, 0.322)	(0.206, 0.228)	(0.345, 0.383)	(0.329, 0.365)	(0.297, 0.330)	(0.234, 0.259)
Increase in price#Loss of income	0.023	0.215	0.203		0.222	0.159	0.144
	(0.017, 0.029)	(0.204, 0.225)	(0.192, 0.213)		(0.211, 0.233)	(0.151, 0.167)	(0.136, 0.151)
Increase in price#Poor				0.414			
				(0.393, 0.434)			
Relied on savings	− 0.132	− 0.104	− 0.092	− 0.327		− 0.171	− 0.053
	(− 0.126, − 0.139)	(− 0.066, − 0.133)	(− 0.087, − 0.096)	(− 0.310, − 0.343)		(− 0.163, − 0.181)	(− 0.051, − 0.056)
Borrowed	0.168	0.544	0.222	0.218		0.119	0.102
	(0.160, 0.176)	(0.517, 0.571)	(0.211, 0.233)	(0.207, 0.23)		(0.113, 0.125)	(0.097, 0.107)

95% CI in parentheses.

**Table 3:** Determinants of food insecurity—went without eating for a whole day.

	(1)	(2)	(3)	(4)	(5)	(6)	(7)	(8)	(9)	(10)
	Armenia	Cambodia	Chad	Djibouti	Ethiopia	Kenya	Mali	Malawi	Nigeria	Uganda
HH head gender (female)	0.159	− 0.116	− 0.116	0.245	0.266	0.254	0.053	0.284	0.117	0.178
	(0.151, 0.167)	(− 0.110, − 0.122)	(− 0.072, − 0.161)	(0.233, 0.257)	(0.253, 0.28)	(0.242, 0.267)	(0.050, 0.056)	(0.270, 0.299)	(0.111, 0.123)	(0.170, 0.188)
HH head years of schooling	− 0.049		− 0.041	− 0.195	− 0.156	− 0.084		− 0.037	− 0.016	− 0.082
	(− 0.046, − 0.051)		(− 0.039, − 0.043)	(− 0.186, − 0.205)	(− 0.148, − 0.164)	(− 0.080, − 0.088)		(− 0.035, − 0.039)	(− 0.016, − 0.017)	(− 0.078, − 0.087)
Income loss	0.216	0.589			− 0.167	0.173		0.113	0.132	0.356
	(0.205, 0.227)	(0.561, 0.619)			(− 0.159, − 0.176)	(0.164, 0.182)		(0.107, 0.119)	(0.125, 0.138)	(0.339, 0.374)
Poor			0.135	0.293			0.228			
			(0.129, 0.142)	(0.279, 0.309)			(0.217, 0.24)			
Safety net (cash)—wave 1				− 0.038	− 0.127			− 0.218	− 0.015	− 0.075
				(− 0.036, − 0.04)	(− 0.121, − 0.133)			(− 0.207, − 0.229)	(− 0.014, − 0.016)	(− 0.073, − 0.077)
Safety net (cash)—wave 2		− 0.183			− 0.218			− 0.287	− 0.091	− 0.123
		(− 0.181, − 0.184)			(− 0.207, − 0.229)			(− 0.273, − 0.301)	(− 0.087, − 0.095)	(− 0.120, − 0.126)
Safety net (cash)—wave 3		− 0.229			− 0.269			− 0.351		− 0.182
		(− 0.226, − 0.232)			(− 0.256, − 0.282)			(− 0.334, − 0.368)		(− 0.179, − 0.185)
Safety net (cash)—wave 4		− 0.270			− 0.294				− 0.146	− 0.247
		(− 0.268, − 0.272)			(− 0.281, − 0.308)				(− 0.140, − 0.152)	(− 0.241, − 0.253)
Safety net (cash)—wave 5					− 0.266					
					(− 0.252, − 0.281)					
Safety net (cash)—wave 6					− 0.232					
					(− 0.22, − 0.243)					
Safety net (cash)—wave 7									− 0.192	
									(− 0.183, − 0.201)	
Safety net (food)—wave 1				0.150	0.321			0.151	0.192	0.168
				(0.143, 0.158)	(0.306, 0.336)			(0.144, 0.158)	(0.182, 0.202)	(0.160, 0.176)
Safety net (food)—wave 2		0.291			0.310			0.142	0.134	0.137
		(0.288, 0.293)			(0.294, 0.325)			(0.135, 0.149)	(0.127, 0.141)	(0.130, 0.144)
Safety net (food)—wave 3		0.201			0.220			0.113		0.104
		(0.198, 0.204)			(0.210, 0.231)			(0.107, 0.119)		(0.099, 0.109)
Safety net (food)—wave 4		0.179			0.202				0.111	0.061
		(0.177, 0.18)			(0.193, 0.212)				(0.106, 0.116)	(0.058, 0.064)
Safety net (food)—wave 5					0.186					
					(0.176, 0.196)					
Safety net (food)—wave 6					0.138					
					(0.131, 0.145)					
Safety net (food)—wave 7									0.085	
									(0.081, 0.091)	
Safety net (overall)—wave 1	− 0.025					0.086				
	(− 0.024, − 0.027)					(0.082, 0.090)				
Safety net (overall)—wave 2						0.044				
						(0.042, 0.046)				
Safety net (overall)—wave 3						− 0.034				
						(− 0.033, − 0.035)				
Increase in price of major food items	0.103	0.241	0.219	0.141	0.136	0.142	0.321	0.307	0.131	0.045
	(0.098, 0.108)	(0.229, 0.253)	(0.209, 0.23)	(0.134, 0.148)	(0.129, 0.143)	(0.135, 0.15)	(0.305, 0.337)	(0.293, 0.324)	(0.124, 0.137)	(0.043, 0.047)
Increase in price#Loss of income	0.257	0.404	0.266		0.322	0.103		0.108	0.144	0.333
	(0.244, 0.271)	(0.384, 0.425)	(0.253, 0.28)	0.304	(0.306, 0.339)	(0.098, 0.108)		(0.103, 0.114)	(0.137, 0.152)	(0.317, 0.350)
Increase in price#Poor				(0.289, 0.320)			0.409			
							(0.389, 0.431)			
Relied on savings	0.039	− 0.031	− 0.172		− 0.015	− 0.091	− 0.251		− 0.048	− 0.095
	(0.037, 0.041)	(− 0.029, − 0.032)	(− 0.163, − 0.181)		(− 0.014, − 0.016)	(− 0.087, − 0.096)	(− 0.239, − 0.264)		(− 0.046, − 0.051)	(− 0.091, − 0.1)
Borrowed	0.429	0.051	0.133		0.684	0.163	0.120		0.080	0.083
	(0.408, 0.451)	(0.049, 0.054)	(0.126, 0.14)		(0.651, 0.720)	(0.155, 0.172)	(0.114, 0.126)		(0.076, 0.084)	(0.079, 0.087)

95% CI in parentheses.

**Table 4:** Determinants of food insecurity—ran out of food.

	(1)	(2)	(3)	(4)	(5)	(6)	(7)	(8)
	Cambodia	Chad	Ethiopia	Mali	Malawi	Nigeria	South Africa	Uganda
HH head gender (female)	0.048	− 0.131	0.212	0.062	0.363	0.249	0.322	0.101
	(0.046, 0.051)	(− 0.125, − 0.138)	(0.202, 0.223)	(0.059, 0.065)	(0.346, 0.381)	(0.237, 0.262)	(0.307, 0.338)	(0.096, 0.106)
HH head years of schooling		− 0.079	− 0.048		− 0.045	− 0.147	− 0.063	− 0.093
		(− 0.075, − 0.083)	(− 0.046, − 0.051)		(− 0.043, − 0.048)	(− 0.140, − 0.155)	(− 0.06, − 0.066)	(− 0.089, − 0.098)
Income loss	0.367		0.143		0.193	0.261	0.274	0.274
	(0.350, 0.386)		(0.136, 0.150)		(0.184, 0.203)	(0.248, 0.274)	(0.261, 0.287)	(0.262, 0.288)
Poor		0.342		0.265				
		(0.326, 0.360)		(0.252, 0.278)				
Safety net (cash)—wave 1			− 0.103		− 0.267	− 0.018	− 0.208	− 0.118
			(− 0.101, − 0.105)		(− 0.264, − 0.270)	(− 0.017, − 0.019)	(− 0.202, − 0.214)	(− 0.112, − 0.124)
Safety net (cash)—wave 2	− 0.145		− 0.178		− 0.344	− 0.088	− 0.266	− 0.125
	(− 0.138, − 0.153)		(− 0.174, − 0.181)		(− 0.339, − 0.349)	(− 0.084, − 0.092)	(− 0.259, − 0.273)	(− 0.118, − 0.132)
Safety net (cash)—wave 3	− 0.199		− 0.241		− 0.424		− 0.313	− 0.155
	(− 0.189, − 0.209)		(− 0.235, − 0.247)		(− 0.420, − 0.428)		(− 0.306, − 0.320)	(− 0.147, − 0.163)
Safety net (cash)—wave 4	− 0.231		− 0.275			− 0.137		− 0.223
	(− 0.219, − 0.242)		(− 0.268, − 0.282)			(− 0.130, − 0.144)		(− 0.212, − 0.234)
Safety net (cash)—wave 5			− 0.252					
			(− 0.247, − 0.257)					
Safety net (cash)—wave 6			− 0.235					
			(− 0.230, − 0.239)			− 0.204		
Safety net (cash)—wave 7						(− 0.193, − 0.215)		
Safety net (food)—wave 1	0.328		0.351		0.113	0.191	0.147	0.111
	(0.311, 0.345)		(0.344, 0.357)		(0.112, 0.114)	(0.183, 0.199)	(0.143, 0.151)	(0.109, 0.111)
Safety net (food)—wave 2	0.208		0.328		0.095	0.144	0.115	0.099
	(0.197, 0.219)		(0.322, 0.335)		(0.094, 0.096)	(0.138, 0.15)	(0.112, 0.118)	(0.098, 0.100)
Safety net (food)—wave 3	0.233		0.233		0.058		0.096	0.064
	(0.221, 0.244)		(0.227, 0.238)		(0.057, 0.059)		(0.094, 0.098)	(0.063, 0.065)
Safety net (food)—wave 4			0.208			0.112		0.051
			(0.203, 0.213)			(0.107, 0.117)		(0.050, 0.052)
Safety net (food)—wave 5			0.190					
			(0.186, 0.193)					
Safety net (food)—wave 6			0.140					
			(0.138, 0.143)					
Safety net (food)—wave 7						0.093		
						(0.089, 0.097)		
Increase in price of major food items	0.256	0.370	0.298	0.105	0.424	0.039	0.119	0.248
	(0.243, 0.268)	(0.353, 0.389)	(0.284, 0.313)	(0.099, 0.110)	(0.404, 0.444)	(0.037, 0.041)	(0.114, 0.124)	(0.236, 0.260)
Increase in price#Loss of income	0.336	0.446	0.154		0.216	0.121	0.309	0.238
	(0.319, 0.353)	(0.424, 0.469)	(0.146, 0.162)		(0.205, 0.227)	(0.115, 0.127)	(0.294, 0.325)	(0.227, 0.25)
Increase in price#Poor				0.107				
				(0.102, 0.112)				
Relied on savings	− 0.041	− 0.105	− 0.098	− 0.008		− 0.026		− 0.212
	(− 0.039, − 0.043)	(− 0.100, − 0.110)	(− 0.093, − 0.103)	(− 0.005, − 0.011)		(− 0.025, − 0.027)		(− 0.202, − 0.223)
Borrowed	0.046	0.126	0.281	0.029		0.031		0.301
	(0.044, 0.049)	(0.122, 0.131)	(0.268, 0.295)	(0.031, 0.028)		(0.029, 0.032)		(0.286, 0.316)

95% CI in parentheses.

**Table 5:** Determinants of food insecurity—went hungry.

	(1)	(2)	(3)	(4)	(5)	(6)	(7)	(8)	(9)	(10)
	Cambodia	Chad	Djibouti	Ethiopia	Kenya	Mali	Malawi	Nigeria	South Africa	Uganda
HH head gender (female)	− 0.080	− 0.178	0.122	0.158	0.298	0.050	0.541	0.022	0.364	0.212
	(− 0.076, − 0.084)	(− 0.170, − 0.187)	(0.116, 0.129)	(0.150, 0.166)	(0.283, 0.314)	(0.047, 0.052)	(0.516, 0.569)	(0.020, 0.023)	(0.346, 0.383)	(0.201, 0.223)
HH head years of schooling		− 0.074	− 0.232	− 0.055	− 0.120		− 0.006	− 0.156	− 0.045	− 0.102
		(− 0.07, − 0.078)	(− 0.221, − 0.244)	(− 0.052, − 0.057)	(− 0.114, − 0.126)		(− 0.005, − 0.007)	(− 0.149, − 0.164)	(− 0.043, − 0.047)	(− 0.097, − 0.108)
Income loss	0.371			0.260	0.257		0.336	0.327	0.338	0.251
	(0.352, 0.391)			(0.247, 0.273)	(0.245, 0.271)		(0.320, 0.354)	(0.310, 0.344)	(0.321, 0.356)	(0.239, 0.264)
Poor		0.309	0.300			0.382				
		(0.293, 0.324)	(0.285, 0.315)			(0.364, 0.402)				− 0.091
Safety net (cash)—wave 1			− 0.046	− 0.083			− 0.226	− 0.083	− 0.129	(− 0.089, − 0.093)
			(− 0.044, − 0.049)	(− 0.081, − 0.086)			(− 0.222, − 0.23)	(− 0.079, − 0.087)	(− 0.123, − 0.135)	− 0.139
Safety net (cash)—wave 2	− 0.126			− 0.153			− 0.269	− 0.149	− 0.163	(− 0.136, − 0.142)
	(− 0.120, − 0.132)			(− 0.149, − 0.157)			(− 0.268, − 0.270)	(− 0.142, − 0.156)	(− 0.155, − 0.171)	− 0.241
Safety net (cash)—wave 3	− 0.226			− 0.211			− 0.311		− 0.227	(− 0.236, − 0.246)
	(− 0.215, − 0.236)			(− 0.204, − 0.218)			(− 0.307, − 0.315)		(− 0.216, − 0.238)	− 0.336
Safety net (cash)—wave 4	− 0.306			− 0.276				− 0.201		(− 0.330, − 0.342)
	(− 0.293, − 0.319)			(− 0.269, − 0.284)				(− 0.191, − 0.211)		
Safety net (cash)—wave 5				− 0.257						
				(− 0.250, − 0.265)						
Safety net (cash)—wave 6				− 0.245						
				(− 0.238, − 0.253)						
Safety net (cash)—wave 7								− 0.295		
								(− 0.281, − 0.309)		
Safety net (food)—wave 1			0.170	0.341			0.133		0.142	0.229
			(0.162, 0.179)	(0.325, 0.358)			(0.130, 0.136)	0.229	(0.134, 0.150)	(0.227, 0.231)
Safety net (food)—wave 2	0.328			0.296			0.107	(0.222, 0.236)	0.111	0.152
	(0.325, 0.331)			(0.282, 0.311)			(0.107, 0.107)	0.185	(0.105, 0.117)	(0.149, 0.155)
Safety net (food)—wave 3	0.229			0.208			0.085	(0.182, 0.192)	0.094	0.116
	(0.227, 0.231)			(0.197, 0.218)			(0.084, 0.086)		(0.089, 0.099)	(0.115, 0.117)
Safety net (food)—wave 4	0.200			0.185				0.124		0.082
	(0.199, 0.201)			(0.176, 0.194)				(0.120, 0.128)		(0.081, 0.083)
Safety net (food)—wave 5				0.163						
				(0.155, 0.171)						
Safety net (food)—wave 6				0.119						
				(0.113, 0.125)						
Safety net (food)—wave 7								0.092		
								(0.089, 0.095)		
Safety net (overall)—wave 1					0.131					
					(0.124, 0.138)					
Safety net (overall)—wave 2					0.095					
					(0.090, 0.100)					
Safety net (overall)—wave 3					− 0.061					
					(− 0.058, − 0.064)					
Increase in price of major food items	0.245	0.307	0.235	0.327	0.247	0.404	0.116	0.184	0.115	0.140
	(0.233, 0.258)	(0.291, 0.323)	(0.223, 0.247)	(0.311, 0.344)	(0.235, 0.260)	(0.384, 0.425)	(0.110, 0.122)	(0.175, 0.193)	(0.109, 0.12)	(0.133, 0.147)
Increase in price#Loss of income	0.214			0.355	0.304		0.266	0.333	0.337	0.363
	(0.203, 0.225)			(0.337, 0.374)	(0.289, 0.320)		(0.253, 0.280)	(0.316, 0.35)	(0.32, 0.355)	(0.345, 0.382)
Increase in price#Poor		0.442	0.405			0.447				
		(0.420, 0.466)	(0.385, 0.426)			(0.426, 0.470)				
Relied on savings	− 0.023	− 0.131		− 0.285	− 0.053	− 0.046		− 0.111		− 0.057
	(− 0.022, − 0.024)	(− 0.125, − 0.138)		(− 0.271, − 0.300)	(− 0.051, − 0.056)	(− 0.044, − 0.049)		(− 0.106, − 0.117)		(− 0.055, − 0.06)
Borrowed	0.104	0.212		0.617	0.133	0.258		0.185		0.089
	(0.099, 0.110)	(0.202, 0.223)		(0.586, 0.649)	(0.126, 0.140)	(0.246, 0.271)		(0.175, 0.194)		(0.084, 0.093)

95% CI in parentheses.

## Discussion

The lower-income countries addressed in this paper have been affected by, and responded to, the COVID-19 pandemic in different and varied ways. However, a number of common patterns emerge from the data that enable us to make several important contributions to the literature addressing food insecurity during the COVID-19 pandemic, and food insecurity in low- and middle-income countries (LMICs) more broadly. First, we have conducted the first multi-country, multi-time period analysis of the key factors influencing food insecurity during the pandemic. Across 11 countries and up to seven waves of data, we show how a combination of the direct health impacts of the pandemic and government responses restricting movement and trade have resulted in food insecurity that government safety nets have not fully mitigated. Our econometric analysis identifies key socioeconomic determinants of food insecurity during the pandemic. Importantly, we quantify these effects and demonstrate heterogeneity across countries and across food insecurity indicators, highlighting the importance of understanding local contexts. Specifically, households that are female-headed, less-educated, poor or experiencing a pandemic-induced loss of income, or without access to savings are more likely to suffer from food insecurity. These findings are consistent with much of the literature on the socioeconomic determinants of food insecurity^22^; and with^23^ who highlight key socioeconomic inequalities in COVID-19 related mortality. Because the educational background of parents influences their children’s educational, earnings, and wage outcomes^24,25^, our results further suggest that public investment and policies to improve education are likely to reduce inter-generational inequalities with respect food insecurity during any future crises, including possible future pandemics.

Second, we also show the differentiated impacts of household coping strategies—specifically savings versus borrowing. Our findings show that households that were able to rely on savings had a lower probability of suffering from food insecurity during the pandemic compared to the households that had to borrow, even controlling for poverty or loss of income. This finding is consistent with the literature that suggests that encouraging lower-income households to save will improve food security for the poor, not just in the context of the pandemic, and can build resilience to shocks and mitigate the more severe negative impacts of those shocks^26–28^. Third, our analysis confirms that the observed increases in the prices of major food items have increased the chances of food insecurity across countries, particularly for relatively poorer households and those that have suffered a loss in income.

Finally, and importantly, our differentiated analysis of the efficacy of safety nets over time and across multiple countries demonstrates definitively that during this pandemic, cash assistance has reduced the chances of households suffering from food insecurity over time, whereas food assistance has failed to significantly reduce the probability of food insecurity during the pandemic. Prior to the pandemic, cash and food assistance have both been shown more broadly to improve food security across various dimensions. However, we recognise that there are other elements of food security, such as nutritional aspects and diet diversity, that we do not address, and that may not be influenced by cash transfers^29^. Many countries (such as Malawi, Mali, Nigeria, and South Africa) extended cash support using pre-pandemic information on vulnerability based on poverty status during the pandemic^30^. Our results support governments designing more targeted policies that take into account local contexts and location specific socioeconomic drivers of food insecurity; and targeting of particularly vulnerable groups including those who may be considered newly vulnerable due to shocks such as the COVID-19 pandemic. Indeed, a number of countries have expanded cash transfers in a more targeted way, such as to the elderly (Cabo Verde, Mauritania), the disabled (Mauritania and Tunisia), and female-headed households (Mauritania). Further, countries including Madagascar and Sudan provided food assistance specifically to those who relied on daily earning who are particularly affected by government lockdown measures^31^. Combining these country specific targeted initiatives with our paper’s empirical findings offers guidance for governments as to how to ensure more effective food security outcomes from limited budgets.

Our paper, one of the most comprehensive pieces of research to address food insecurity quantitatively across multiple countries and timescales, has revealed important empirical regularities of relevance to those responsible for improving food and nutrition status in households across lower-income countries. Our research supports the need for appropriately targeted safety nets, designed with an understanding of the specific country and household characteristics, and a broad understanding of food security that takes into account the importance of quality and nutritional content, in addition to quantity.

We recognise that though the data sets we use for our analysis are nationally representative, they are not directly comparable across countries and time, for example, in terms of the number of waves conducted, nor do they control for any potential seasonality effects. That said, we do control for the wave fixed-effects, and so do pick up seasonality aspects through the time fixed-effects. Furthermore, we do not run a pooled regression, instead the econometric analysis is conducted separately for each country, and therefore the risk of seasonality affecting our results is minimal.

These important debates over how to best reduce food insecurity tend to flare up during crises, such as pandemics, but are increasingly relevant more broadly given the persistence of food insecurity across LMICs and growing evidence that climate change is resulting in an increase in the number and proportion of under-nourished people across the globe^32^. As evidence increases that climate change is directly harming food security, government policies will be central to how countries adapt to the realities of climate change, particularly in terms of the likely increases in frequency and severity of shocks, on top of the slow-burn impact of increasing temperatures on crop yields.

## Supplementary Information

Supplementary Information.

## Data Availability

The data used in this paper are publicly available at https://microdata.worldbank.org/index.php/catalog/hfps.

## Data statement

All methods were carried out in accordance with relevant guidelines and regulations. We use secondary data for our analysis. The surveys were conducted by national statistical offices of the respective countries, who obtained informed consent from all the respondents. The datasets used were anonymised by removing all identifying information on households and individuals before being made available for research purposes.
