# Supplementary material for: Impact of COVID-19 on food insecurity using multiple waves of high frequency household surveys
Source: Sci Rep. 2022 Feb 3;12:1865. doi: 10.1038/s41598-022-05664-3 (PMC8814158; doi:10.1038/s41598-022-05664-3)
Supplement: Supplementary file 1 — Supplementary Information. [file 41598_2022_5664_MOESM1_ESM.pdf]

Supplementary information -Impact of COVID-19 on food insecurity using multiple waves of high frequency household surveys

Appendix A

Cases, deaths, and government restrictions

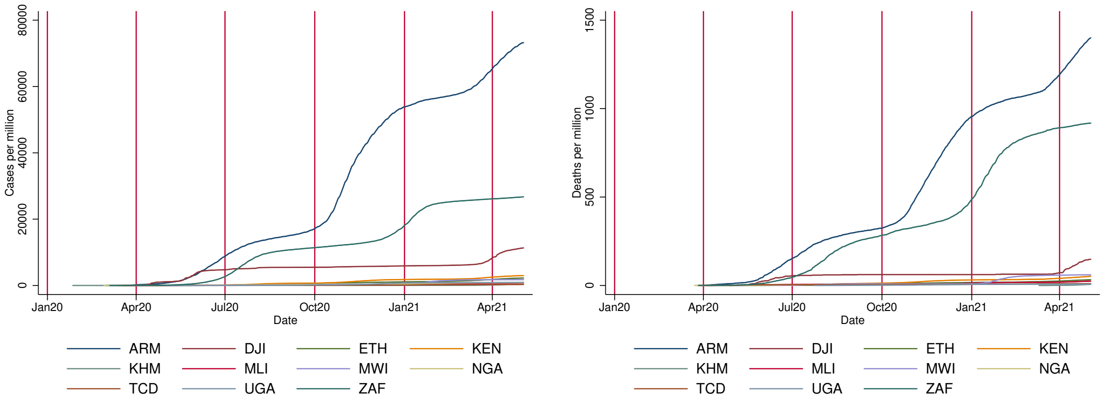

Figure 1: Reported COVID-19 cases and deaths

Safety nets

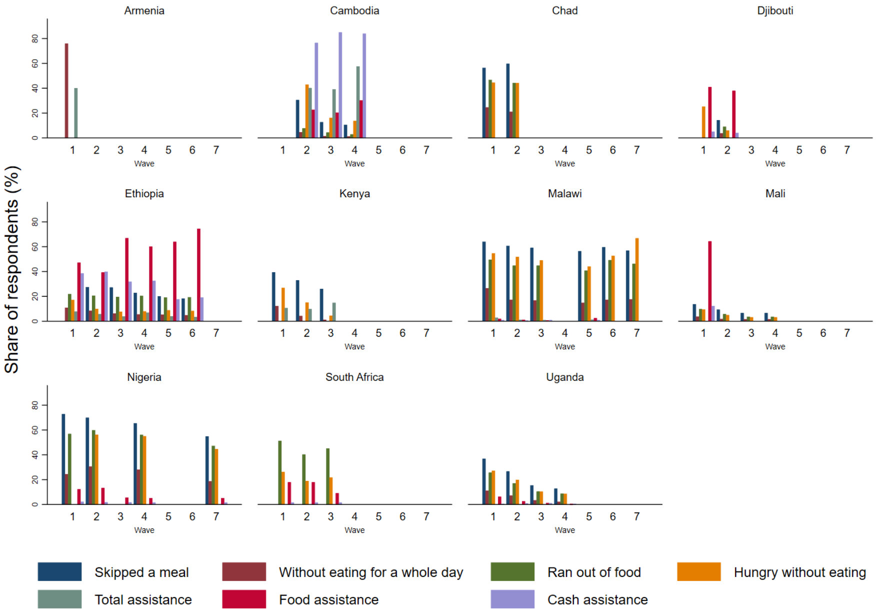

Figure 2: Tracking food insecurity and safety nets during the pandemic

Food insecurity at the sub-national level

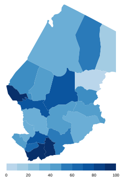

(a) Skipped a meal - wave 1

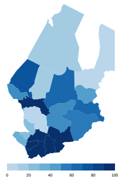

(b) Skipped a meal - wave 3

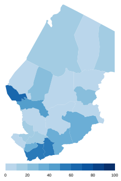

(c) Whole day - wave 1

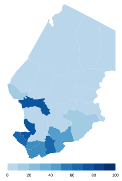

(d) Whole day - wave 3

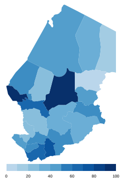

(e) Ran out food - wave 1

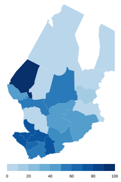

(f) Ran out food - wave 3

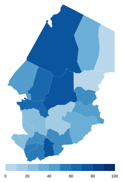

(g) Went hungry - wave 1

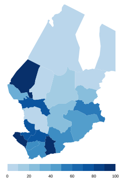

(h) Went hungry - wave 3

Figure 3: Chad - share of households

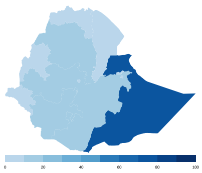

(a) Whole day - wave 1

(b) Whole day - wave 6

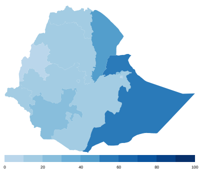

(c) Run out of food - wave 1

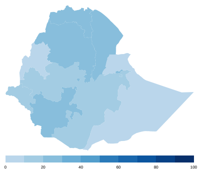

(d) Run out of food - wave 6

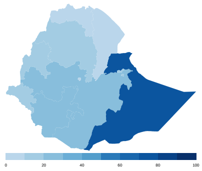

(e) Went hungry - wave 1

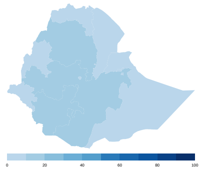

(f) Went hungry - wave 6

Figure 4: Ethiopia - share of households

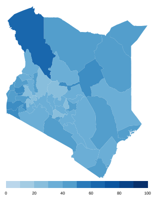

(a) Skipped a meal - wave 1

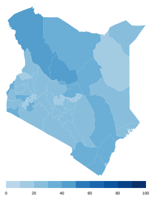

(b) Skipped a meal - wave 3

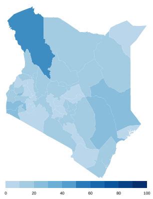

(c) Whole day - wave 1

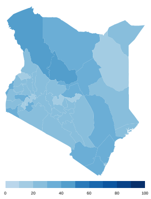

(d) Whole day - wave 3

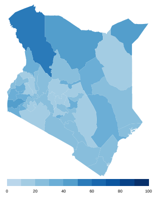

(e) Ran out food - wave 1

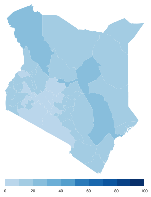

(f) Ran out food - wave 3

Figure 5: Kenya - share of households

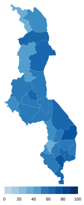

(a) Skipped a meal - wave 1

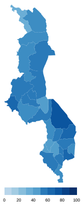

(b) Skipped a meal - wave 7

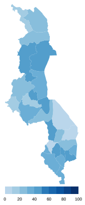

(c) Whole day - wave 1

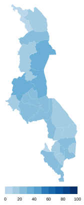

(d) Whole day - wave 7

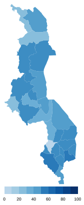

(e) Ran out food - wave 1

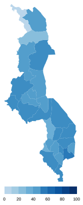

(f) Ran out food - wave 7

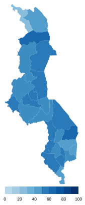

(g) Went hungry - wave 1

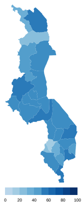

(h) Went hungry - wave 7

Figure 6: Malawi - share of households

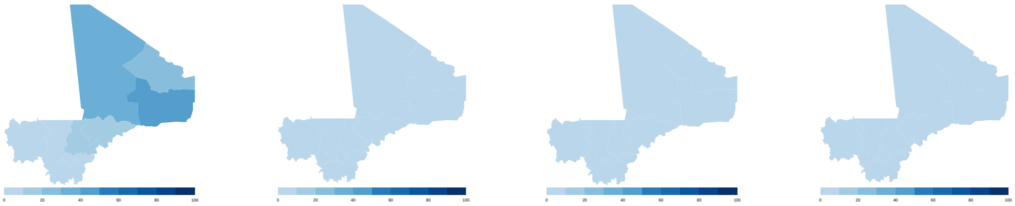

(a) Skipped a meal - wave 1 (b) Skipped a meal - wave 4 (c) Whole day - wave 1 (d) Whole day - wave 4

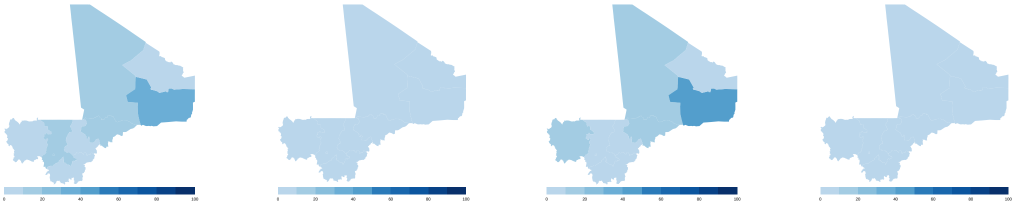

(e) Ran out food - wave 1 (f) Ran out food - wave 4 (g) Went hungry - wave 1 (h) Went hungry - wave 4

Figure 7: Mali - share of households

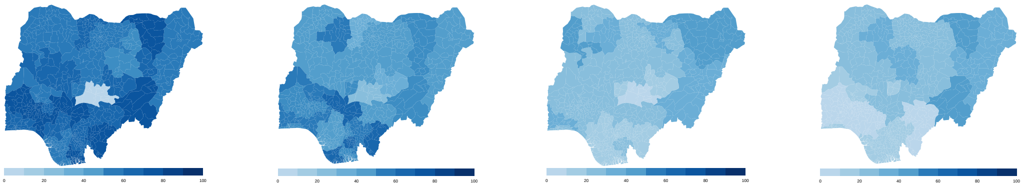

(a) Skipped a meal - wave 1 (b) Skipped a meal - wave 7 (c) Whole day - wave 1 (d) Whole day - wave 1

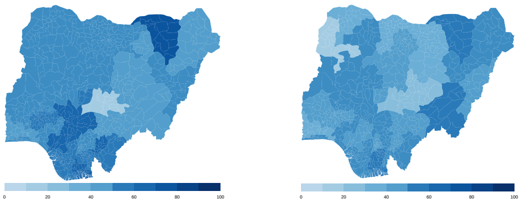

(e) Ran out food - wave 1 (f) Ran out food - wave 7

Figure 8: Nigeria - share of households

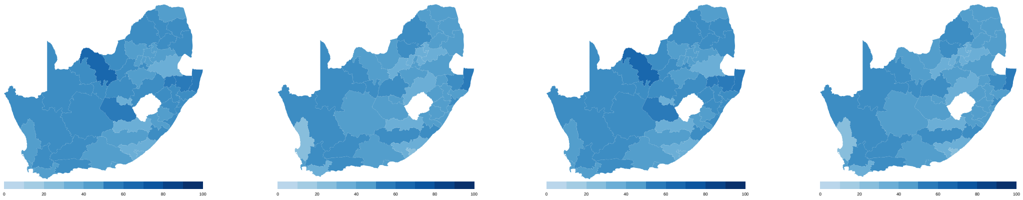

(a) Ran out food - wave 1 (b) Ran out food - wave 3 (c) Went hungry - wave 1 (d) Went hungry - wave 3

Figure 9: South Africa - share of households

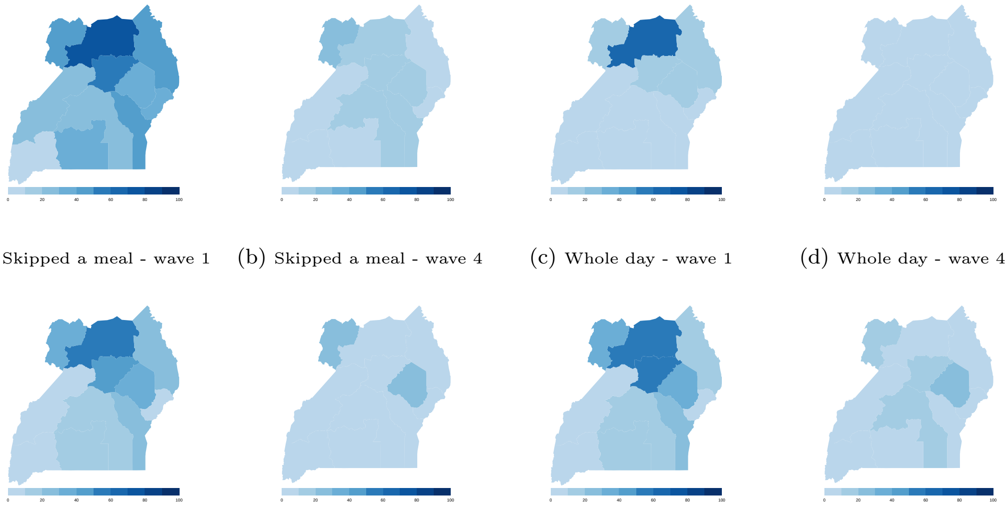

(a) Skipped a meal - wave 1 (b) Skipped a meal - wave 4 (c) Whole day - wave 1 (d) Whole day - wave 4

(e) Ran out food - wave 1 (f) Ran out food - wave 4 (g) Went hungry - wave 1 (h) Went hungry - wave 4

Figure 10: Uganda - share of households

Figures 3-10: The maps show the food insecurity indicators at the sub-national level for each country and wave. We aggregate the household-level responses to the food (in)security related questions to sub-national regions of the corresponding countries using the survey weights provided. We use the command *spplot* from the *sp* package (version 1.4-6) in R (version 4.1.2).
